# Assessing Frequency and Appropriateness of Proton Pump Inhibitor Deprescription in Patients Requiring Endoscopic Therapy for Esophageal Strictures

**DOI:** 10.1093/jcag/gwad036

**Published:** 2023-09-30

**Authors:** Kevin L Kecskemeti, Mark Borgaonkar, Jerry McGrath

**Affiliations:** Faculty of Medicine, Memorial University of Newfoundland, St. John’s, Canada; Faculty of Medicine, Memorial University of Newfoundland, St. John’s, Canada; Faculty of Medicine, Memorial University of Newfoundland, St. John’s, Canada

## Abstract

**Objective:**

There have been concerns about the widespread usage of proton pump inhibitors (PPIs), leading to recommendations to deprescribe PPIs in certain patients. This study aims to determine if PPI deprescription in patients with symptomatic esophageal strictures was consistent with published guidelines and to compare the rate of PPI deprescription between two-time points.

**Methods:**

All patients from two gastroenterology practices who received endoscopic dilation to treat symptomatic strictures between the years of 2015–2017 and 2019–2021 were identified using physician billing codes. We defined inappropriate PPI deprescription as: a patient who was deprescribed their PPI with a past medical history of esophageal stricture, Barrett’s esophagus, grade C/D esophagitis, or who had experienced symptom recurrence after PPI deprescription. Furthermore, we analyzed the rate of PPI deprescription between two time periods 2015–2017 (group 1) and 2019–2021 (group 2).

**Results:**

Two hundred twenty-three esophageal dilations were analyzed. Twenty-six patients in the sample were deprescribed their PPI, with the majority (57 percent) meeting the criteria for inappropriate PPI deprescription. There was a trend towards more inappropriate deprescription in the second time period. (71 percent vs. 33 percent; *P* = 0.06). Patients in group 2 had a higher rate of PPI deprescription (23.9 percent) than group 1 (6.0 percent; *P* < 0.001).

**Conclusions:**

PPI deprescription in patients treated for symptomatic esophageal strictures appears to be more common in the second time period. Most patients were defined as inappropriate deprescription. Physicians must apply guidelines carefully when considering deprescribing PPIs.

## Introduction

Proton pump inhibitor (PPI) usage has become widespread since omeprazole was first introduced to the market in 1989.^1^ It is estimated that between 40 percent and 55 percent of primary care patients in the United States are prescribed a PPI without a clear indication.^2^ Although PPIs are well tolerated and considered to be safe, their widespread usage is not without controversy. Concerns regarding polypharmacy, rising healthcare costs, and adverse events such as impaired B-12 absorption^3^, hip fractures^4^, pneumonia^5^, dementia^6^, and *Clostridium difficile* infection^7^ have been reported in patients using PPIs in observational studies.^3–7^ These concerns have encouraged PPI deprescription in select patients with low-medium risk GERD.

The ACG guidelines for the management of gastroesophageal reflux disease(GERD) recommend that patients with LA grades C and D esophagitis require lifelong PPI therapy to maintain healing.^8^ Likewise, the American Gastroenterological Association states in their clinical practice guidelines on deprescribing PPIs that patients with a history of severe esophagitis and peptic stricture should generally not be considered for PPI discontinuation.^9^ We wanted to assess how common PPI deprescription was at our centre for patients who required esophageal dilation for strictures and to determine the frequency of inappropriate deprescription.

## Methodology

This retrospective cohort study analyzed patients who received esophageal dilation for strictures between the years of 2015–2017 (group 1) and 2019–2021 (group 2). We selected these dates as they corresponded with the release of PPI deprescription guidelines. In March 2018, Choosing Wisely Canada (CWC) disseminated guidelines for primary care physicians encouraging PPI deprescription in low-medium risk patients with GERD.^10^ Although the CWC guidelines were not mandatory, the organization provided recommendations to physicians with the goal of reducing unnecessary treatments.^11^ Our study aimed to compare the rate of PPI deprescription across these two time periods. We received local HREB approval for this study on February 1, 2022.

All patients from two academic gastroenterology practices who received endoscopic dilation to treat symptomatic esophageal strictures during these years were identified using physician billing codes. We aimed to reduce selection bias by identifying all patients requiring esophageal dilation from two gastroenterology practices as opposed to using a smaller patient subset from a single practice. Once the patients were identified we conducted a chart review using the Meditech electronic medical database. We analyzed endoscopy reports, narrative reports, and medication forms for the following patient information: sex, age, date of dilation, type of dilation, presence of pre-existing upper gastrointestinal diagnosis, diagnosis following dilation, previous PPI medication dose and frequency, and current PPI medication dose and frequency. All the patient information was coded using a standardized data sheet and entered into SPSS for data analysis.

We defined PPI deprescription as either a 50 percent dose reduction, frequency reduction, or complete medication discontinuation at the time of endoscopic dilation compared to the established PPI therapy from the previous 3 months or longer. Next, we defined inappropriate PPI deprescription as a patient who was deprescribed their PPI with a past history of esophageal stricture, Barrett’s esophagus, grade C/D esophagitis, or who had experienced symptom recurrence after PPI deprescription. We selected these criteria as they are consistent with both Canadian and American Gastroenterology Society GERD management guidelines for conditions requiring long-term acid suppression therapy.

For our sample size calculation, we estimated a deprescription rate of 5 percent for group 1 and 20 percent for group 2. Using an alpha value of 0.05 and a beta value of 0.20, we required a sample size of at least seventy-two patients in each group.

## Results and data analysis

### Patient inclusion and exclusion criteria

We identified 246 dilations between the years of 2015–2017 and 2019–2021. One patient with imatinib esophagitis, who had no drug coverage for a PPI, and who had eleven dilations during the study period was excluded. Furthermore, a total of twelve patients were excluded from the study because of incomplete medical records. These patients did not have an endoscopy report or narrative report describing their esophageal dilation or PPI prescription history. In total twenty-three dilations were excluded, leaving 223 esophageal dilations to be assessed from 183 unique patients.

### Patient demographics and characteristics

Demographic data were collected from the 223 esophageal dilations and 183 unique patients. The average age of the sample was 57.6 ± 15.9 years old. The sample consisted of 105 males (57 percent) and 78 females (42 percent). Patient diagnosis following endoscopic dilation is listed in Table 1. The most common diagnosis in our sample was GERD at 50 percent (*n* = 112), followed by eosinophilic esophagitis at 9 percent. The PPI used by patients at the time of endoscopy is documented in Supplementary Tables. The most common PPI in our sample was rabeprazole sodium (sixty-seven patients). In total, fifty-six patients were not taking a PPI at the time of endoscopic dilation. Of these patients, forty-one had newly diagnosed GERD or had non-reflux causes for stricture. The remaining fifteen patients had completely discontinued their PPI.

**Table 1.** Diagnosis after endoscopy.

Diagnosis		Percentage (%)
GERD	112	50.2
Eosinophilic esophagitis	21	9.4
Radiation or chemotherapy	12	5.4
Schatzki’s ring	7	3.1
Normal endoscopy	7	3.1
Barrett’s esophagus	6	2.7
Ulcer disease	4	1.8
Eophageal lichen planus	2	0.9
Cricopharyngeal bar	2	0.9
Other	10	5.4
Total	183	100

Other include: Crohn’s disease, achalasia, muscular dystrophy, pill-induced esophagitis, esophageal web, and stricture secondary to previous surgery.

In our sample, twenty-six unique patients were deprescribed their PPI. Their demographics, diagnosis at the time of dilation and type of deprescription are listed in Table 2. The most common event was complete PPI discontinuation in fifteen patients. Fifty-seven percent (15/26 cases) of patients met our criteria for inappropriate PPI deprescription (Table 3).

**Table 2.** Demographic characteristics of the twenty-six deprescribed patients.

Age	59.4 ± 14.2	
Gender	M:16 F:10	
Diagnosis	Number	Percentage (%)
GERD	22	85
Schatzki’s ring	2	7.7
Esophageal web	1	3.8
Radiation/Chemotherapy	1	3.8
Total	26	100
Type of PPI deprescription	Number	Percentage (%)
Complete PPI discontinuation	15	57.8
Frequency reduction	8	30.7
Dose reduction	2	7.8
Dose and frequency reduction	1	3.8
Total	26	100

**Table 3.** Inappropriate PPI deprescription.

Previous stricture	10/26
Barrett’s esophagus	0/26
Grade C/D esophagitis	1/26
Symptom recurrence	4/26
Total inappropriate deprescription:	15/26 = 57%
Total appropriate deprescription (no PMH):	11/26 = 43%

### PPI deprescription rates across time

There were twice as many dilations in group 1 (*n* = 151) compared to group 2 (*n* = 72). This was due to a sabbatical during the second time period and the overall reduction in procedure volumes during the initial phases of the coronavirus disease 2019 (COVID-19) pandemic. There were no statistically significant differences in the demographic characteristics of the two groups (Table 4).

**Table 4.** Demographic information between groups.

	Group 1 (2015–2017)	Group 2 (2019-2021)	*P*
Number of patients	151	72	
Mean age	59.0 ± 15.9	58.1 ± 15.6	0.670
Sex (M:F)	80:71	44:28	0.253
Percent of patients with GERD diagnosis following dilation	84/151 = 56%	48/72 = 67%	0.09

In total, twenty-six patients were identified who were deprescribed their PPIs. The patients receiving dilations from group 1 (2015–2017) had a PPI deprescription rate of 6.0 percent (9/151 cases). Meanwhile, patients from group 2 (2019–2021) had a PPI deprescription rate of 23.9 percent (17/72 cases) (*P* = 0.003) (Table 5).

**Table 5.** Chi-squared of PPI deprescription rates for the entire cohort and GERD patients.

Chi-squared	PPI deprescribed	PPI Non-deprescribed	Total
Group 1	9	142	151
Group 2	17	55	72
Total	26	197	223
Chi-squared	PPI deprescribed	PPI Non-deprescribed	Total
Group 1	7	77	84
Group 2	15	33	48
Total	22	110	132

Fisher Exact test: *P* value = 0.0003 for the entire cohort.

Fisher Exact test: *P* value = 0.0012 for GERD patient subgroup.

Patients were further subdivided based on their diagnosis following endoscopic dilation. We identified 132 patients whose primary diagnosis was GERD. Patients with GERD from group 1 had a PPI deprescription rate of 8.4 percent (7/84 cases). Meanwhile, patients with GERD from group 2 had a PPI deprescription rate of 31 percent (15/48 cases) (*P* = 0.0012).

### Assessing inappropriate PPI deprescription between the two groups

The 26 patients with PPI deprescription had an average length of 14 months between PPI deprescription and esophageal stricture dilation. In group 1, 33 percent (3/9) of the deprescribed patients were defined as inappropriate. While, in group 2, 71 percent (12/17) were defined as inappropriate. Overall, there was a trend towards more inappropriate deprescription in the second time period (*P* = 0.06).

## Discussion

There are a variety of reasons why patients on long-term PPIs should be considered for deprescription. Some may not have a clear indication for PPIs, while others may have mild GERD that requires only episodic therapy. However, patients with more severe GERD are generally felt to require uninterrupted PPI therapy, especially those with severe esophagitis, Barrett’s esophagus, and peptic stricture.

In our study, we observed that PPI deprescription was more common in 2019–2021 compared to 2015–2017. As well, more than half the patients deprescribed were defined as inappropriate (57 percent) with a trend towards more inappropriate deprescriptions in the second time period (2019–2021). There are many possible explanations for the observation of greater PPI deprescription in the later time period, including concern about PPI safety and recommendations to use PPIs only for patients with a clear indication and reduced access to physician and healthcare services, especially during the COVID-19 pandemic.

Recommendations to consider PPI deprescription occurred in our province in 2018 and may have influenced PPI prescribing and patient adherence. These guidelines were meant to be applied to low and medium-risk patients with uncomplicated GERD. By identifying patients who were inappropriately deprescribed, we may have observed instances where guidelines were applied incorrectly. Clinical guidelines are powerful tools, but their utility is dependent on physicians and healthcare workers applying them appropriately to the correct patient population. In our study, there were cases of patients with clear medical indications for long-term acid suppression who had their PPIs deprescribed.

Other researchers have analyzed the impact of PPI deprescription in patients with GERD and noted that many patients report worsening symptoms.^12^ In a randomized trial of patients with GERD, Björnsonn et al.^13^ found that only 21 percent of patients were able to successfully discontinue PPI therapy during the 12-month study due to symptom recurrence. This is also recognized in the 2022 ACG guidelines that recommend that patients with GERD who require maintenance therapy should use PPIs at the “lowest dose that controls symptoms and maintains healing of reflux esophagitis.” Furthermore, these guidelines acknowledge that abrupt PPI deprescription can lead to rebound acid hypersecretion and increased reflux symptoms.

The risks of PPI therapy have been studied extensively in observational studies.^3–7^ However, a randomized controlled trial of 17,500 patients found that only enteric infection was more common in patients who received pantoprazole compared to placebo.^14^ The authors found the number needed to harm is larger than 300 patients with 3 years of PPI usage, suggesting that for most patients with reflux disease the benefits of therapy likely outweigh the risks.

Our study has many limitations. It consists of a small sample from only two gastroenterologists at a single centre. It was conducted retrospectively such that not all relevant data could be collected for each patient. By selecting all patients treated for esophageal strictures, we had a heterogeneous group, although we did perform subgroup analysis on patients with GERD. We did not measure esophageal acid exposure between groups or for patients before and after PPI deprescription. We recruited all patients who had endoscopic dilation for esophageal strictures during the study years but there were no standardized criteria to assess for the severity of symptoms necessitating dilation. Furthermore, there may be other unique factors to our healthcare centre and our province that may limit the generalizability of our findings to other jurisdictions.

In conclusion, we noted that more patients receiving esophageal dilation in 2019–2021 had had PPI deprescription than in 2015–2017. A subset of those patients had deprescription that would be considered inappropriate based on published guidelines. We speculate that a number of factors, including poor specialist access during the COVID-19 pandemic and guidelines encouraging PPI deprescription, may have contributed to this finding. However, more research in this area is needed, particularly in larger samples in other jurisdictions to help corroborate our findings.

## Supplementary Material

gwad036_suppl_Supplementary_Tables

## Data Availability

The data underlying this article will be shared on reasonable request to the corresponding author.
