# Supplementary material for: Assessing Frequency and Appropriateness of Proton Pump Inhibitor Deprescription in Patients Requiring Endoscopic Therapy for Esophageal Strictures
Source: J Can Assoc Gastroenterol. 2023 Sep 30;6(6):229–33. doi: 10.1093/jcag/gwad036 (PMC10723932; doi:10.1093/jcag/gwad036)
Supplement: gwad036_suppl_Supplementary_Tables [file gwad036_suppl_supplementary_tables.docx]

**Supplemental figures**

Table 6: Proton pump inhibitor medication at the time of endoscopic dilation

| *Medication* | Frequency | *Proportion* | Percentage |
| --- | --- | --- | --- |
| Pariet | 67 | 67/223 | 30.0 |
| Pantoloc | 17 | 17/223 | 7.6 |
| Tecta | 30 | 30/223 | 13.5 |
| Losec | 19 | 19/223 | 8.5 |
| Prevacid | 2 | 2/223 | 0.9 |
| Nexium | 17 | 17/223 | 7.6 |
| Dexilant | 15 | 15/223 | 6.7 |
| No PPI | 56 | 56/223 | 25.1 |
| Total | 223 | 223/223 | 100.0 |

Table 7: PPI medication, dose, and frequency at the time of endoscopic dilation

| PPI Frequency | PPI Dose (mg) | Pariet | Pantoloc | Tecta | Losec | Prevacid | Nexium | Dexilant |
| --- | --- | --- | --- | --- | --- | --- | --- | --- |
| OD | 10 | 1 | 0 | 0 | 0 | 0 | 0 | 0 |
|  | 20 | 55 | 4 | 5 | 11 | 0 | 4 | 0 |
|  | 30 | 0 | 0 | 0 | 0 | 2 | 0 | 2 |
|  | 40 | 0 | 6 | 18 | 0 | 0 | 6 | 0 |
|  | 60 | 0 | 0 | 0 | 0 | 0 | 0 | 11 |
| BID | 10 | 0 | 0 | 0 | 0 | 0 | 0 | 0 |
|  | 20 | 8 | 1 | 1 | 6 | 0 | 2 | 1 |
|  | 30 | 0 | 0 | 0 | 0 | 0 | 0 | 0 |
|  | 40 | 1 | 4 | 5 | 0 | 0 | 5 | 0 |
|  | 60 | 0 | 0 | 0 | 0 | 0 | 0 | 0 |
| TID | 10 | 0 | 0 | 0 | 0 | 0 | 0 | 0 |
|  | 20 | 0 | 0 | 0 | 0 | 0 | 0 | 0 |
|  | 30 | 0 | 0 | 0 | 0 | 0 | 0 | 0 |
|  | 40 | 0 | 1 | 0 | 0 | 0 | 0 | 0 |
|  | 60 | 0 | 0 | 0 | 0 | 0 | 0 | 0 |
| PRN | 10 | 0 | 0 | 0 | 0 | 0 | 0 | 0 |
|  | 20 | 2 | 0 | 0 | 2 | 0 | 0 | 0 |
|  | 30 | 0 | 0 | 0 | 0 | 0 | 0 | 0 |
|  | 40 | 0 | 1 | 1 | 0 | 0 | 0 | 0 |
|  | 60 | 0 | 0 | 0 | 0 | 0 | 0 | 0 |

Definitions: OD (once daily), BID (twice daily), TID (three times daily) and PRN (as needed).
